# Psychological Health and Digital Social Participation of the Older Adults during the COVID-19 Pandemic in Blekinge, Sweden—An Exploratory Study

**DOI:** 10.3390/ijerph19063711

**Published:** 2022-03-21

**Authors:** Sarah Nauman Ghazi, Peter Anderberg, Johan Sanmartin Berglund, Jessica Berner, Ana Luiza Dallora

**Affiliations:** 1 Department of Health, Blekinge Institute of Technology, SE-371 79 Karlskrona, Sweden; peter.anderberg@bth.se (P.A.); johan.sanmartin.berglund@bth.se (J.S.B.); jessica.berner@bth.se (J.B.); ana.luiza.moraes@bth.se (A.L.D.); 2 School of Health Sciences, University of Skövde, SE-541 28 Skövde, Sweden

**Keywords:** gerontology, gerontechnology, aging, digital social participation, psychological health, COVID-19, public health, eHealth

## Abstract

COVID-19 has affected the psychological health of older adults directly and indirectly through recommendations of social distancing and isolation. Using the internet or digital tools to participate in society, one might mitigate the effects of COVID-19 on psychological health. This study explores the social participation of older adults through internet use as a social platform during COVID-19 and its relationship with various psychological health aspects. In this study, we used the survey as a research method, and we collected data through telephonic interviews; and online and paper-based questionnaires. The results showed an association of digital social participation with age and feeling lack of company. Furthermore, in addition, to the increase in internet use in older adults in Sweden during COVID-19, we conclude that digital social participation is essential to maintain psychological health in older adults.

## 1. Introduction and Background

Health is not just the well-being of the body but also the well-being of our mind [1]. Several life stressors can affect this, such as losing loved ones, political scenarios, warfare, and pandemics. The pandemics of infectious diseases affecting people’s psychological health are widely known. Some studies even imply that the population’s frequency of mental health problems due to communicable diseases exceeds that of its physical impact [2]. The COVID-19 pandemic, apart from its severe physical impact, has posed various challenges in terms of psychological health. The World Health Organisation has shown concerns about the pandemic’s impact on mental health and psycho-social repercussions [3]. It has pointed out some factors like isolation and quarantine that may increase the prevalence of mental health conditions such as anxiety or stress, loneliness, depression, insomnia and, even obsessive-compulsive disorder (OCD) [4]. Prevalence of insomnia [5], disturbance in appetite [6] and worrying excessively for oneself, family members, or loved ones contracting COVID-19 were also reported [7]. Other than that, loneliness in terms of missing company of friends and family and feeling socially distanced might affect the psychological health of the older adults [8]. Although the pandemic has affected all age groups, older adults are the most affected group in this pandemic [9] due to weak immune systems and aging-related health conditions [10,11].

In Sweden, older adults (aged 65 and above) are around 20 percent of the population [12]. Out of 15,015 deaths in Sweden due to COVID-19, 14,452 deaths are of individuals aged 60 years which is 96.2% of the total deaths as of 3 November 2021 [13].

To prevent the spread of COVID-19 among the population in general and especially among the older adults, the recommendation of maintaining social distance, staying home, avoiding public and social gatherings and being in isolation when there are similar symptoms to COVID-19, were proposed by the public health agency [14].

Social participation, an essential modifiable determinant of health [15], has no universally accepted definition. Most of the definitions in the literature are centered on the involvement of a person in activities that provide interactions with others in the society or community [16]. The internet is one of the mediums that can provide the platform for interactions within the society or community. Thus came the term Digital participation or Digital Social Participation (DSP) which means ‘active involvement in digital society through the use of modern information and communication technology (ICT), such as the Internet’ [17]. Contrary to those mentioned above, social disconnectedness, isolation, and quarantine are risks of increasing non-participation and anxiety and depression [18]. Encouraging the use of ICT may play a valuable role during the pandemic to decrease social disconnectedness. Older adults have a lesser tendency to use the internet than younger adults. In Sweden, only 62.4% of the individuals above 65 are internet users [19]. This increases the risk of a digital divide between the young and the old as they tend to use the internet more [20]. Older adults who continue to face their health deteriorating by the time and adding new challenges every passing year, the digital divide can worsen their independence and social connectedness [21]. Following the onset of the COVID-19 pandemic, internet use to stay connected with loved ones has increased. This communication is seen as a coping mechanism for stress due to COVID-19 [22]. The increase in internet usage and digital social participation has reduced social isolation and loneliness in older adults [23,24].

Furthermore, the vulnerability to death and fear due to COVID-19 impact the psychological health of older adults contributing to stress and anxiety [25]. The psychological distress in the older Swedish population due to COVID-19 may also be seen as collateral damage of COVID-19 [26]. Psychological health is defined as being able to use technology, which leads to longer, active, and more meaningful lives as well as more active daily mental function [27,28]. In this study, psychological health aspects which are considered in respect to Covid-19 are the factors such as low mood, anxiety, insomnia, low appetite, worry about themselves and family contracting COVID-19, feeling socially distanced and missing company of family and friends.

Considering the previous research conducted on COVID-19 and older adults, the authors would like to investigate social participation and psychological health in this pandemic [29].

Therefore, this study aims to explore:

1. The factors that affect the psychological health in relation to digital social participation (DSP) in older adults in Sweden during the COVID-19 pandemic.
2. The sample’s socio-demographic(SD) characteristics associated with digital social participation.

## 2. Method

The study presented herein follows a cross-sectional survey as the research methodology.

### 2.1. Study Population

The study population is composed of 740 participants from the age of 65 years and older. This study uses the data collected from the Swedish National Study on Aging and Care in Blekinge (SNAC-B), a longitudinal research project in Sweden. SNAC-B is a part of the larger study SNAC that aims to establish longitudinal databases which will provide a better understanding of the process of aging [30].

During the first outbreak of COVID-19 in August 2020, the participants were interviewed through telephone, emailed, or mailed, according to their preferences. The survey was specifically designed for COVID-19 and all the questions were asked in context of the ongoing pandemic. Introduction to the survey stated in Swedish “We are now conducting a survey in SNAC regarding the COVID 19 pandemic on health and social life”. The purpose of the questionnaire was to investigate the impact of COVID-19 on physical health, psychological health, daily activities, and digital social participation.

The inclusion criteria consisted of all the older adults above 65 who are part of the SNAC-B study. The exclusion criteria were lack of ability to communicate due to frail condition, high cognitive impairment or language barrier. As a result, 584 replied out of 740, corresponding to a response rate of 78.9%. Out of 157 non-respondents, 57 did not want to participate, 26 had passed away, 19 were not reachable, and 55 were too sick to participate or enrolled in care facilities.

### 2.2. Measures

Psychological health in COVID-19 was assessed through eight questions that are the factors for disturbing psychological health. Four questions from the Comprehensive Psychopathological Rating Scale (CPRS) pool, two about being worried about themselves and their family contracting COVID-19. Two items of loneliness in terms of missing company of friends and family, and feeling socially distanced during COVID-19 were also included in survey.

The CPRS serves as a pool from which required items can be selected to fulfill the purpose of the investigation [31]. These questions aimed to investigate the following psychological aspects: Low Mood, Anxiety, Insomnia, and low Appetite. They were dichotomized ‘Yes’ or ‘No’, where ‘No’ corresponds to a score of zero i.e., the absence of the symptoms. The presence of a symptoms was labeled as ‘Yes’ (with score 1 or more. The dichotomization of CPRS items had been carried out in previous studies where they were dichotomized into the presence and absence of a symptom [32]).

The other four factors that can affect psychological health during COVID-19 are worrying about oneself and loved ones being affected by COVID-19, feeling a lack of company, and being socially distanced during the pandemic. All these items were dichotomized into ‘No’ and ‘Yes’ for analysis, where ‘No’ were the ones not affected and ‘yes’ were the affected ones in the COVID-19.

Digital social participation (DSP) during COVID-19 was assessed using an instrument, Social Score, to measure the social participation of older adults regarding their use of the internet as a social platform. Social Score, developed by Anderberg et al. [33], is a 6 item Likert scale measuring DSP and coherence with the society through digital tools. It has a Cronbach (α) coefficient of 0.9 1 in our study.

The total score of the participants ranges from 6-30. In this study, the total social score is presented as a mean score (1–5) to depict the weighted average of each item in the total score. The higher the score the better the digital social participation. For logistic regression, DSP variable was categorized as Low (score 1.0–2.5) and High (score 2.6–5.0). The DSP questions were only asked from those who use the internet.

Age was used as a continuous and categorical variable divided into three groups (65–74; 75–84; 85 + years ) regarding the socio-demographic variables. Gender was used as a binary variable. The Swedish old education system, which is relevant to our participants’ age, was divided into three groups: low, middle, and high level of education. The economic status was measured as a binary variable. The participants were asked a yes or no if they could get up to 14,000 SEK in a week if an unpredicted expense arrived. They were marked as a good economic status with a yes, and no was marked as a poor economic status. At the time of data collection in 2020, this was a typical and non-invasive question used in the SNAC study to assess the economic status. The living arrangement information was also collected as living alone or with someone. Health status was asked on a Likert scale of 1–7 (much better to much worse in COVID-19). During the analysis, this health status was trichotomized into Good, Neutral (neither good nor poor), and Poor. Internet use was inquired as a yes or no question and was analyzed as a binary variable.

### 2.3. Statistical Analysis

The data was analyzed using STATA version 16.1 (developed by STATACorp LLC, College Station, TX, USA). Statistical analysis includes univariate analysis of sociodemographic variables, psychological health variables, and digital social participation scores. ‘Don’t know’ and the missing data for the variables were omitted during the analysis as they constituted less than 5% of the sample.

The Shapiro-Wilk test was done to check the normality of the data. An inferential Mann-Whitney test was used to determine a statistically significant difference in the DSP score among the dichotomized psychological health variables. The same test was done for the socio-demographic variable consisting of two groups. However, for age groups, education, and health status, the Kruskal Wallis test was used to determine differences between the variables and DSP. Finally, logistic regression was performed to examine the association between DSP (dependent variable) and significant variables from Mann-Whitney and Kruskal Wallis test. The significance value was set to 0.05.

### 2.4. Ethical Consideration

Written informed consent was collected from all the participants of the SNAC-B. They were briefed that they have the right to withdraw from the study at any point and that their anonymity will be respected. This study is approved by the Research Ethics committee of Lund University (LU 604-00).

## 3. Results

### 3.1. Descriptive Analysis

In this survey, a total of 584 responses (78.91%) were collected from the ages of 65 to 100. In Table 1, we present the sample characteristics. The mean age of the respondents was 77.4 years where 53.42% identified themselves as females. As for the educational qualification, most respondents (45.6%) attended secondary school and 27.47% attended elementary school. Whereas, 26.92% of the participants reported to have acquired higher education. We also asked the participants about their household economy. A majority of the participants have a good economic status (92.15%) compared to 7.85% who reported a poor household economy. It was identified that only 35.45% of the participants lived alone compared to 64.55% who responded otherwise. The respondents’ perceived health status corresponded majorly (75.47%) to the perception that their health is neither good nor poor(neutral), while only 5.7% felt they had good and 18.83% felt poor health status. Internet use corresponded to 71.92% who use the internet while 28.08% do not. The Shapiro-Wilk test of normality showed non-normal distribution of data with *p*-value < 0.0001.

Table 2, presents the results of the psychological health data collected from the participants. The results show that 30.93% of the participants experienced low/depressive moods. Moreover, 32.68% of the participants reported anxiety and 31.81% experienced insomnia. Only 34.27% of the respondents reported bad appetite during the COVID-19 pandemic. Further, 41.25% reported to be more worried about family members being affected by COVID-19 and 24.18% were worried about themselves that they might get affected by COVID-19. Also, 52.80% of people felt socially distanced and 56.70% suffered a lack of company in COVID-19.

For digital social participation in the pandemic, 394 out of 415 internet users (94.93%) older adults responded to the social score questions. The total score of each participant ranges from 6–30. The mean total score is 17.92 (Range: 6–30, SD: 7.43). The mean social score of each participant ranges from 1–5.The sample mean of DSP indicated a high score of 2.98 (SD:1.24). The distribution of DSP scores among the variables are shown in Table 3 and Table 4.

### 3.2. Socio-Demographic Variables and Digital Social Participation

Mann-Whitney and Kruskal-Wallis tests were performed to check significant difference in the DSP score among the socio-demographic variables. A Kruskal-Wallis test showed a statistically significant difference in DSP score between the different age groups, ${\tilde{\chi }}^{2}$(2) = 6.17, *p* = 0.04, with a mean DSP score of 3.04 for 65–74, 3.04 for 75–84, and 2.57 for 85+ age group. The detailed results of the statistical tests performed are presented in Table 3.

### 3.3. Psychological Health Variables and Digital Social Participation

A statistically significant difference in DSP score was found between the groups who feel a lack of company of their friends and family during COVID-19 and those who do not (z = 2.009, *p* = 0.04). Results of the test are presented in Table 4.

Lower DSP scores were noticed among those who felt a lack of company ($\bar{x}$:2.86, SD (σ):1.22) versus those who did not feel a lack of company ($\bar{x}$:3.12, SD (σ): 1.24).

### 3.4. Logistic Regression

The significant variables from the univariate analyses were entered into a logistic regression with the digital social participation as the dependent variable, and the independent variables were age groups (65–74, 75–84, 85+)and feeling lack of company. The results indicate no significant relationship between age groups and digital social participation. On the other hand, digital social participation has statistically significant difference in individuals feeling lack of company during COVID-19 to those not feeling otherwise. (OR = 0.62 *; 95% CI: 0.41–0.94). Table 5 shows logistic regression of significant variables, their odds ratio, 95% CI and *p*-value.

## 4. Discussion

This exploratory study aims to investigate more thoroughly the nature of digital social participation (DSP) and psychological health during the COVID-19 pandemic [34].

The main findings of this study are:

- The feeling of lack of company is significantly related to more digital social participation.
- Age is significantly associated with digital social participation.

In this study, the DSP score instrument by Anderberg et al. was used to explore the association between digital social participation with psychological health and socio-demographic variables. Although this instrument was validated through comparing it with existing instruments, Anderberg et al. [33] suggest in their study that this instrument should be validated in other contexts. In our study, the DSP score instrument has been applied for the first time in the context of psychological health, which needs further investigation of the validity of the instrument in other contexts.

The psychological variable feeling lack of company was significantly related to digital social participation, where there is a lesser likelihood of higher DSP. This means that feeling lack of company during COVID-19 is negatively associated with DSP. Increasing DSP might help in fulfilling the need of company of family and friends [35].

In addition to that, our univariate results show that DSP is significantly less in higher age groups of 85+ years than younger age groups. In order to increase digital social participation in the oldest older adults, a suggestion would be the involvement of this age group when creating platforms that address activities relevant to their lives and peers [36]. This is important to avoid technophobia among older adults and should be addressed to reduce social isolation and improve quality of life [21,37].

In our study, other socio-demographic factors like gender, education, living alone or not are not significantly associated with digital social participation. Although insignificant, we do not have enough evidence to state the non-existence of association between variables and DSP [38] and that there is a minor difference seen during descriptive analysis and it agrees to the studies done previously. Males and females have different frequencies of participation in a digital society, where females tend to be more socially active using a digital platform [39,40]. In addition, higher education was a strong predictor of internet use [41] and thus became associated with high DSP. In line with the previous study [42], cohabiting individuals are more likely to use the internet and participate digitally than those living alone. This is probably due to encouragement or assistance provided by cohabiting family members, friends, or partners [42].

We considered eight aspects as distress factors in psychological health. Four of these aspects are from the pool of CPRS, two aspects about the individuals being worried for themselves and their family whereas the final two aspects are in the context of loneliness considering the missing company of their friends and family and feeling socially distanced during COVID-19.

This study shows that the participants were evidently in psychological distress during the Covid-19 in Blekinge. The most dominant factors were being worried about family contracting COVID-19 [20] and loneliness, i.e., feeling lack of company and socially distanced in the COVID-19 [43]. Due to social distancing and isolation, social meetings with family and friends were minimized, which led to one of the main psychosocial problems during the COVID-19, i.e., loneliness [43].

This study indicated that participating in the digital society will enable the people feel less isolated. enabled more contact with other people. A significant negative association was found between the presence of loneliness and DSP score [24]. A higher DSP lowers the feeling lack of company of friends and family [23] due to the pandemic. Other studies have also indicated that digital participation has a mitigating effect on loneliness [44].

Although we could not find any significant association between other psychological health factors and DSP, we noticed some emerging patterns. Individuals that were worried about contracting COVID-19 for themselves and their loved ones, had a high level of DSP. As per the recommendations, the older adults were required to maintain isolation and social distance during the pandemic. This social deprivation could have led them to worry more about their family and friends than about contracting the disease themselves. These individuals might have found an alternative in the digital sphere to stay connected with their family and friends during the pandemic, which is visible through a high DSP score.

Internet use has increased during the pandemic [45]. Previously, internet use among older adults, in Blekinge, before COVID-19 showed the frequency 62% [19]. However, during the pandemic, our data from the same population show that the frequency of internet use in older adults in Blekinge is 71.92%, which is a substantial increase. This is in line with the results from another recent study, which also evidenced that internet use has significantly increased following the onset of the pandemic and as a coping mechanism for stress [22].

## 5. Limitations of the Study

One of the limitations of this study is that the study population is from mid-sized and rural cities and did not include large-sized cities. However, the large-sized cities had the strongest outbreak, so this could impact the results.

In this study, the variable household economy is not representative of the population and it is skewed towards the positive side.

Further, there is an instrument limitation. We obtained only certain items from the pool of CPRS that were relevant for our study during the recent pandemic combined with other psychological health aspects important during the COVID-19 like being worried for themselves and family or friends and lack of company, and social isolation.

Moreover, this study type is cross sectional and thus causality between DSP and psychological health during the pandemic cannot be ascertained.

## 6. Conclusions

The COVID-19 pandemic has the potential of affecting the psychological health of older people, which could result in psychosocial consequences like the feeling of lack of company and thus, loneliness. Internet use and digital social participation potentially assist in alleviating these feelings. This study investigated associations between digital social participation and psychological health that need to be further explored in future studies. Future studies should investigate how the pandemic has impacted the use of the internet and what types of internet use the older adults have and would like to engage in.

## Figures and Tables

**Table 1:** Characteristics of the sample (N = 548) in terms of socio-demographic variables and digital social participation.

Age mean (SD)		77.4 (7.75)
Age n (%)	65–74 years	249 (42.64)
	75–84 years	214 (36.64)
	85+ years	121 (20.72)
Gender n (%)	Male	272 (46.58)
	Female	312 (53.42)
Education n (%)	Elementary school	150 (27.47)
	Secondary school	249 (45.60)
	Higher education	147 (26.92)
Living arrangement n (%)	Alone	207 (35.45)
	Not alone	377 (64.55)
Household economy n (%)	Poor	41 (7.85)
	Good	481 (92.15)
Health status n (%)	Good	33 (5.70)
	Neutral	437 (75.47)
	Poor	109 (18.83)
Internet users n (%)	Yes	415 (71.92)
	No	162 (28.08)
Digital social participation score n (%)		394 (94.93%)
Digital social participation score mean (SD)(Range)		2.98 (1.23) (1–5)

**Table 2:** Characteristics of the sample in terms of psychological health variables.

Comprehensive Psychopathological Rating Scale (CPRS) and Other Psychological Health Variables during COVID-19	Yes/No	n (%)
Low mood	No	373 (69.07)
	Yes	167 (30.93)
Anxiety	No	375 (67.32)
	Yes	182 (32.68)
Insomnia	No	388 (68.19)
	Yes	181 (31.81)
Low appetite	No	374 (65.73)
	Yes	195 (34.27)
Worried about contracting COVID-19	No	439 (75.82)
	Yes	140 (24.18)
Worried about family members contracting COVID-19	No	339 (58.75)
	Yes	238 (41.25)
Feeling lack of company	No	249 (43.30)
	Yes	326 (56.70)
Feeling socially distanced	No	270 (47.20)
	Yes	302 (52.80)

**Table 3:** Digital social participation score among the socio-demographic variables in a study sample of n = 394 and *p*-value from Mann-Whitney test/Kruskal Wallis test.

Socio-Demographic Variables		DSP Scores Mean (SD)	(*p*-Value)
Age group	65–74 year	3.04 (1.22)	
	75–84 years	3.04 (1.23)	0.04
	85+ years	2.57 (1.24)	
Gender	Male	2.9 (1.20)	
	Female	3.04 (1.26)	0.34
Education	Elementary school	2.9 (1.51)	
	Secondary school	2.89 (1.22)	0.25
	Higher education	3.09 (1.12)	
Living arrangement	Alone	2.85 (1.19)	
	Not alone	3.05 (1.25)	0.17
Household economy	Poor	3.2 (1.17)	
	Good	2.9 (1.23)	0.35
Health status	Good	3.1 (1.17)	
	Neutral	3.0 (1.25)	0.64
	Poor	2.8 (1.16)	

**Table 4:** Digital social participation score among the CPRS and other psychological health variables in a study sample of n = 394 and *p*-value from Mann-Whitney test.

CPRS and Other Psychological Health Variables during COVID-19	Yes/No	DSP Score Mean (SD)	(*p*-Value)
Low mood	No	3.03 (1.24)	
	Yes	2.87 (1.16)	0.26
Anxiety	No	2.98 (1.28))	
	Yes	3.00 (1.12))	0.93
Insomnia	No	3.01(1.25)	
	Yes	2.94 (1.18)	0.58
Low appetite	No	2.95 (1.22)	
	Yes	3.05 (1.27)	0.53
Worried about contracting COVID-19	No	2.9 (1.26)	
	Yes	3.1 (1.15)	0.45
Worried about family members contracting COVID-19	No	2.9 (1.25)	
	Yes	3.02 (1.22)	0.48
Feeling lack of company	No	3.12 (1.24)	
	Yes	2.86 (1.22)	0.04
Feeling socially distanced	No	3.05 (1.30)	
	Yes	2.9 (1.17)	0.261

**Table 5:** Logistic regression with digital social participation (Low = 0, High = 1) as a dependent (Y) and age and lack of company as independent variables.

Significant Variables		Coefficient	Odds Ratio	95% CI	*p*-Value
Age	65–74 years	Ref			
	75–84 years	−0.14	0.86	0.55–1.35	0.53
	85+ years	−0.47	0.61	0.33–1.15	0.13
Feeling lack of company	No	Ref.			
	Yes	−0.46	0.62	0.41 −0.94	0.02

## Data Availability

The Department of health at Blekinge Institute of Technology (BTH), Sweden, has the datasets of this study archived.
